# Space-time clusters of breast cancer using residential histories: A Danish case–control study

**DOI:** 10.1186/1471-2407-14-255

**Published:** 2014-04-11

**Authors:** Rikke Baastrup Nordsborg, Jaymie R Meliker, Annette Kjær Ersbøll, Geoffrey M Jacquez, Aslak Harbo Poulsen, Ole Raaschou-Nielsen

**Affiliations:** 1 Danish Cancer Society Research Center, Copenhagen, Denmark; 2 National Institute of Public Health, University of Southern Denmark, Copenhagen, Denmark; 3 Graduate Program in Public Health and Department of Preventive Medicine, Stony Brook University, Stony Brook, NY, USA; 4 BioMedware Inc, Ann Arbor, MI, USA; 5 Department of Geography, State University of New York at Buffalo, Buffalo, NY, USA

**Keywords:** Space-time cluster analysis, Breast cancer, Residential histories, *Q*-statistics, Denmark

## Abstract

**Background:**

A large proportion of breast cancer cases are thought related to environmental factors. Identification of specific geographical areas with high risk (clusters) may give clues to potential environmental risk factors. The aim of this study was to investigate whether clusters of breast cancer existed in space and time in Denmark, using 33 years of residential histories.

**Methods:**

We conducted a population-based case–control study of 3138 female cases from the Danish Cancer Registry, diagnosed with breast cancer in 2003 and two independent control groups of 3138 women each, randomly selected from the Civil Registration System. Residential addresses of cases and controls from 1971 to 2003 were collected from the Civil Registration System and geo-coded. *Q*-statistics were used to identify space-time clusters of breast cancer. All analyses were carried out with both control groups, and for 66% of the study population we also conducted analyses adjusted for individual reproductive factors and area-level socioeconomic indicators.

**Results:**

In the crude analyses a cluster in the northern suburbs of Copenhagen was consistently found throughout the study period (1971–2003) with both control groups. When analyses were adjusted for individual reproductive factors and area-level socioeconomic indicators, the cluster area became smaller and less evident.

**Conclusions:**

The breast cancer cluster area that persisted after adjustment might be explained by factors that were not accounted for such as alcohol consumption and use of hormone replacement therapy. However, we cannot exclude environmental pollutants as a contributing cause, but no pollutants specific to this area seem obvious.

## Background

With more than one million new cases each year, breast cancer is the most common cancer among women, accounting for one-fifth of all new female cancer cases worldwide [1]. The industrialised parts of the World have experienced a fast increase in breast cancer incidence during the last decades and still have high incidence rates. Low rates, on the other hand, are found in most Asian and African countries; although incidence rates are also rapidly increasing in these areas [2]. In Denmark the age standardised incidence rate (world standard population) doubled from 46.1 per 100.000 person-years in 1960 to 102.5 in 2010 [3].

The majority of the established breast cancer risk factors are related to oestrogens. Early menarche and late menopause increase the risk, while reproductive factors such as many child births and young age at first birth reduce the risk. Hormone replacement therapy (HRT) for menopause, ionising radiation, alcohol intake, night shift work and some specific genetic mutations are also established risk factors [4-9]. Further, high socioeconomic status is associated with increased risk [10].

Migrant studies of breast cancer found that women, who migrate from areas of low risk to areas of high risk, adopt the higher risk in the host country within a few generations [11,12], and a large study of Scandinavian twins estimated that only 27% of the breast cancer risk was explained by heritable factors [13]. Therefore environmental factors are thought to play a substantial role in the development of breast cancer. Further, a study in the USA estimated that only 41% of the US breast cancer cases were attributable to established risk factors (late age at first birth, nulliparity, family history of breast cancer and high socioeconomic status) [14], leaving the majority of cases unexplained. Persistent organic pollutants such as PCB (polychlorinated biphenyl) and DDT (dichlorodiphenyltrichloroethane) have frequently been studied as environmental risk factors for breast cancer, while the effect of cadmium, electromagnetic fields and solar radiation has been examined to a lesser extent; however, the majority of the studies do not find associations with breast cancer [6,15]. A number of previous studies have used disease mapping and spatial analyses in the search for environmental factors that could be related to breast cancer, however many of these studies relied on aggregated data and used space-only approaches by only including one location to record health events e.g. place of residence at date of diagnosis or date of death [16,17]. However, chronic diseases such as cancer develop over long time, thus causative exposure could occur many years prior to disease manifestation and during that time, individuals may have moved to new addresses several times. Therefore, it is crucial to take human mobility into account in the search for cancer clusters. A study in Western New York applied cluster analyses at selected points in time over the life-course of the study population and identified clustering of breast cancer cases based on place of residence at time of birth and at menarche [18,19]. Whereas, a recent study continuously analyzed breast cancer risk through space and time and found a cluster of breast cancer near a military reservation on Upper Cape Cod, Massachusetts in the 1940s and 1950s [20]. However, only few spatial analyses have led to new hypotheses about environmental risk factors related to breast cancer, perhaps because the majority of the studies neglect human mobility. The aim of this large population-based exploratory study was to investigate if clusters of breast cancer existed in space and time in Denmark, using 33 years of residential histories and accounting for reproductive and socioeconomic factors.

## Methods

### Ethics statement

The Danish Data Protection Agency (2007-41-0437) approved the study. In accordance with Danish law written consent was not obtained as the study was entirely register-based and did not involve biological samples from, or contact with study participants.

### Cases

Female breast cancer cases were identified in the virtually complete population-based Danish Cancer Registry, to which it has been mandatory to report all new cancer diagnoses since 1987 [21]. We included all women diagnosed in 2003 with diagnosis code 170 according to the 7^th^ Revision of the International Classification of Diseases. Only primary cancers were included, however previous diagnoses of non-melanoma skin cancer were allowed. The study included 3138 cases in total.

### Controls

Female controls were randomly selected from the Danish Civil Registration System [22] using incidence density sampling and individually matched with cases by date of birth. Further, controls were alive, living in Denmark and with no previous cancer diagnosis (except from non-melanoma skin cancer) at the date of diagnosis of the matched case. We selected two independent control groups with 3138 women in each group. The selection was carried out with replacement. The purpose of this design was to investigate whether we were able to replicate our findings based on one control group with a second independent group of controls.

### Residential histories

We used the unique personal identification numbers of cases and controls to trace residential histories from 1971 to date of diagnosis of cases and index date of their matched controls by record linkage with the Danish Civil Registration System. Recording of residential data in the civil registration system was not complete before 1971; hence this year was used as the cut-off point. We identified 45,916 unique addresses, each with a unique identification number composed of a municipality code, a road code, and a house number. The dates of moving in and leaving each residence were registered. The addresses were then linked to a register of all official addresses in Denmark, resulting in geographical coordinates for 45,404 of the residential addresses, and missing coordinates for the last 512 (1%) addresses. In the geocoding procedure, 86% of the addresses of both cases and controls matched to the exact house. Four percent matched to one of the neighbouring houses, 2% matched to the centre of the road, and 7% matched at the municipality level, which means that centroid coordinates of the municipality were assigned to these addresses. Equal proportions of addresses of cases and controls were geocoded in each of these categories. The ages of cases and their matched controls were calculated at the beginning and end of each residence, which enabled us to use different time scales in the spatio-temporal cluster analyses [23].

### Covariates

We obtained reproductive data for all cases and controls born in 1935 and onwards using record linkage of the personal identification numbers of cases and controls to the Medical Birth Register [24] and the Danish Family Relations Database, which is based on kinship links between all persons registered in the Danish Civil Registration System [25]. The maternal linkage in the Danish Civil Registration System is considered complete and correct for women born in 1935 and later. Thus, reproductive data were not available for one-third of the study population (1060 cases and 2 sets of 1060 controls) as they were born before 1935. The reproductive data included information on number of live births and age at first live birth. Only children born before date of diagnosis of cases and index date of their matched controls were considered. If there was no information on children in the registers, the women (born in 1935 and onwards) were regarded nulliparous.

From Statistics Denmark we obtained information on socioeconomic indicators aggregated in a 100 meter x 100 meter grid cell net covering all addresses in Denmark. Cells contained average values on income and education in 2008 for a minimum of 100 households at the time. The income variable was based on the yearly disposable household income, while the educational level was based on the person with the highest education in each household. These area-level aggregated measures of income and education were linked with cases and controls based on their most recent residential address.

### Q-statistics

We used *Q*-statistics in the software called SpaceStat (BioMedware Inc., Ann Arbor, MI) to investigate potential space-time clusters of breast cancer. The method has been extensively described in previous studies [23,26,27]. Briefly, this novel approach takes all locations over the entire life-course into account in the cluster analysis. The spatial and temporal local case–control cluster statistic is given in Equation 1:

(1) $$Q_{i,t}^{\left(k\right)}=c_{i}\sum_{j=1}^{k}\eta_{i,j,t}^{\left(k\right)}c_{j}$$

Where for individuals *i* and *j*, *c*_*i*_ and *c*_*j*_ are defined to be 1 if and only if a case, and 0 otherwise. The term $\eta_{i,j,t}^{\left(k\right)}$ is a binary spatial proximity metric that is 1 when participant *j* is a *k* nearest neighbour at time *t* of participant *i*; otherwise it is 0. $Q_{{}_{i,t}}^{\left(k\right)}$ can take on a range of values from 0 to *k* based on the fact that an individual can have up to *k* unique nearest neighbours. This statistic is recalculated for each case every time there is a change in place of residence. In the present study we also calculated $Q_{{}_{i}}^{\left(k\right)}$, which is the sum of each individual’s $Q_{{}_{i,t}}^{\left(k\right)}$ values. This statistic identifies which individuals tend to be centers of clusters over their life-course, while $Q_{{}_{i,t}}^{\left(k\right)}$ determines when and where an individual is a center of a local cluster. We used these two measures in combination to identify when individuals with significant clustering over their life-course co-occurred in space and time. The value of *k* is specified by the user; however there is no standard method to determine the optimal value of *k*.

The statistical significance of the *Q*-statistics was determined by randomly assigning the case–control status to the residential histories under the null hypothesis of no association between places of residence and case–control status. Monte Carlo simulations were used to generate the distributions for hypothesis testing, and the randomization procedure was repeated over 999 iterations, resulting in a minimum *p*-value of 0.001.

For simplicity, we leave out the superscript ^(*k)*^ in the reminder of the paper, but it is understood that the value of the statistic depends on the specification of *k*. Thus $Q_{{}_{i,t}}^{\left(k\right)}$ (the local statistic) is written *Q*_*it*_ and $Q_{{}_{i}}^{\left(k\right)}$ (the subject specific life-course statistic) is written *Q*_*i*_.

Recently, our group conducted a simulation study to evaluate the performance of *Q*-statistics given the propensity for multiple testing and to explore the sensitivity of results to the choice of *k* nearest neighbours [27]. Based on a Danish case–control dataset of similar size as the present study, the simulation study indicated that a *k* of 15 performed well and served as a good starting point. It was also found that a cluster could be further evaluated as a possible true cluster if four or more significant cases were detected in the same area with a *Q*_*i*_*p* = 0.001 and *Q*_*it*_*p* ≤ 0.05 [27]. We used these guidelines in the present study, and performed the first set of analyses with *k* = 15. Subsequently, more analyses were carried out with *k* = 25, 35, 50, and 100.

### Adjustment for covariates

To account for geographical variations in known breast cancer risk factors that may cause clusters, we performed a conditional logistic regression analysis to obtain risk estimates for the association between reproductive and socioeconomic factors and risk of breast cancer. Based on existing knowledge on breast cancer risk factors and data availability we included child birth (ever/never), age at first child birth (continuous), number of children (continuous), area income (continuous) and area education level (continuous) in the model. The risk estimates were then converted into probabilities that a location would be assigned case status as a function of the reproductive and socioeconomic factors. These probabilities were used for adjustment in the spatio-temporal analyses [28]. Consequently, clusters identified in the adjusted analysis would not be attributable to geographical variation in the modelled risk factors.

### Analyses

We ran both unadjusted and adjusted analyses and all analyses were conducted twice, first with control group 1 and then with control group 2. Calendar year and age were applied as two different underlying time scales. Finally, we analyzed data with the two control groups combined in a 1:2 individually matched design. We repeated selected analyses of potential clusters in SaTScan (version 9.1.1) [28,29]. These analyses were conducted on subsets of the original space-time data, with only one location per individual representing time periods with statistically significant clusters identified by *Q*-statistics. We used a Bernoulli model in SaTScan, and the *p*-value for test of significance was obtained from Monte Carlo simulations (999 replications). We analysed elliptical clusters with a maximum cluster size of 15% of the total population and with the option “No Cluster Centers in Other Clusters”.

For clusters that were consistently found across both control groups and in several analyses, we calculated the relative risk of breast cancer associated with a residential history (minimum 5 years) inside the cluster area. Further, we examined if age and extent of tumour at date of diagnosis were different for cases living inside versus outside the cluster areas.

## Results

The study included 3138 cases of breast cancer and two independent control groups with 3138 controls in each. The average age at diagnosis for cases was 63 years, and both cases and controls lived at 4.8 addresses, on average, during the period 1971–2003. Reproductive and socioeconomic data were available for 2078 of the cases and 4155 of the controls corresponding to 66% of the study population; descriptive statistics are summarized in Table 1. The statistics in Table 1 indicate that breast cancer cases had fewer children, were slightly older when they had their first child and that they were living in areas with higher socioeconomic status.

**Table 1:** Descriptive statistics of breast cancer cases and matched controls by factors used for adjustment

**Descriptive statistics**		**Cases**	**Controls**	
Full data set		3138	6276	
Women with missing data on adjustment variables		1060	2121	
Women with data on adjustment variables		2078	4155	
Age at diagnosis/index date^a^		62.6 (41.5 - 85.9)	62.6 (41.5 - 85.9)	
**Adjustment variables**				*p*-value
Child birth yes/no		1838/240 (88%/12%)	3734 / 421 (90%/10%)	0.09^d^
Number of children				0.01^e^
	0	240 (12%)	421 (10%%)	-
	1	328 (16%)	623 (15%)	-
	2	953 (46%)	1910 (46%)	-
	≥3	557 (27%)	1201 (29%)	-
Age at first child birth^a,b^		24.1 (18.6 - 33.5)	23.6 (18.3 - 32.7)	0.0004^e^
Area-level education^a,c^		6.5 (1–31)	5.9 (1–28.2)	0.007^e^
Area-level income in 100.000 DKK^a^		4.9 (2.9 - 7.6)	4.8 (2.8 - 7.4)	0.052^e^

^a^Numbers are medians (5% - 95% percentiles).

^b^Among parous women.

^c^Percentage of households in the area having the highest possible educational level.

DKK: the Danish currency.

^d^Univariate χ^2^-test of categorical variable.

^e^Univariate Wilcoxon test of linear variables.

### Space-time clusters

**Figure 1:**
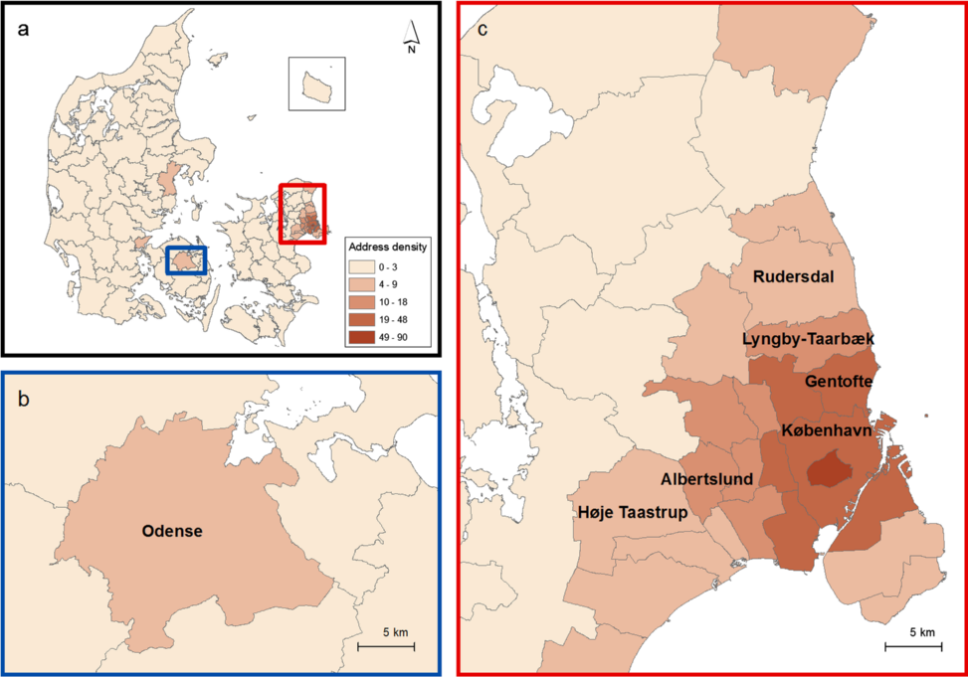
**Overview map of the study area.1a** shows the 98 municipalities of Denmark with two boxes indicating areas where clusters were detected. **1b** shows an enlargement of the Odense area. **1c** shows an enlargement of the Copenhagen area with names of the municipalities referred to in the text. The colours indicate for each municipality the density (number of addresses per square kilometre) of geo-coded residential addresses included in the study. The maps contain data from the Danish Geodata Agency.

Figure 1a shows an overview map of the Danish municipalities, with two boxes indicating areas where clusters were detected: the Odense area (Figure 1b) and the Copenhagen area (the capital of Denmark Figure 1c). Further; these maps show the density (number of addresses per square kilometre) of the study population in each of the 98 Danish municipalities. Overall, clusters were detected in three different areas of Denmark: northern Copenhagen, Odense and Høje Taastrup (south-west of Copenhagen), however; results differed depending on control group, choice of *k*, size of study population and adjustment for covariates.

**Figure 2:**
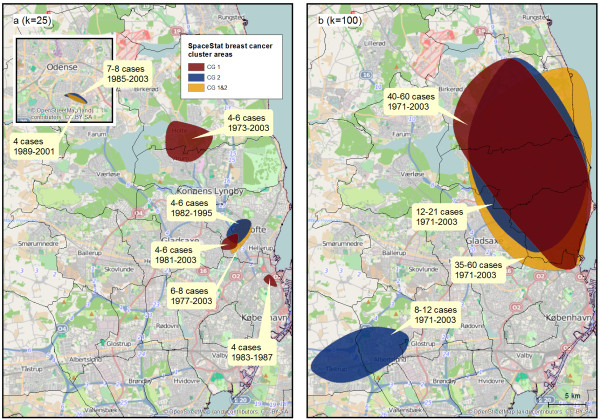
**Results of unadjusted space-time cluster analyses performed in SpaceStat.** Analyses were carried out with 999 permutations, *k* =25 and 100. **2a** shows cluster areas detected at *k* = 25 in the Odense (inserted map) and Copenhagen areas with each of the two control groups as well as when control groups were combined. **2b** shows cluster areas detected at *k* = 100 with each of the two control groups and the combined control group. The cluster areas presented in the figures illustrate the maximum extent of the cluster areas based on the location of significant cases, and the colours of the areas indicate the control group used. This presentation of results secures the anonymity of the study participants (in contrast to presenting the actual address points on the maps). For each cluster area the text box show how many cases it comprised and its temporal extent. CG: Control Group. The maps contain data from the Danish Geodata Agency and © OpenStreetMap (and) contributors, CC- BY-SA.

Figure 2 shows statistically significant breast cancer clusters identified by unadjusted space-time cluster analyses (*Q*-statistics) using *k* = 25 (Figure 2a) and *k* = 100 (Figure 2b) and with calendar year as time scale. With *k* = 25 both control groups and the combined group detected a small cluster north of Copenhagen during the 1980s and 1990s (Figure 2a). Further, control group 1 indentified a cluster north of Copenhagen persisting throughout the study period and a short-term cluster in the city of Copenhagen (red areas in Figure 2a). The second and the combined control groups found a cluster in Odense lasting for about 15 years (blue and yellow areas in Figure 2a). When *k* was increased to 100 both control groups and the combined control group found a larger cluster area with up to 50 cases north of Copenhagen persisting for the whole study period (Figure 2b). Another cluster was identified in the Høje Taastrup area; however only when control group 2 was used (blue area in Figure 2b).

With age as the underlying time scale, application of each of the control groups identified clusters in the area north of Copenhagen at several levels of *k*, also when the control groups were combined. The cluster areas existed when participants were in their 40s to 60s and in the same area (results not shown) as detected when calendar year was applied.

**Figure 3:**
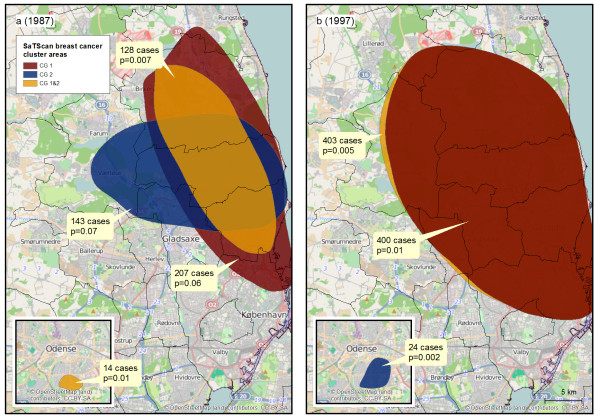
**Results of space-only cluster analyses performed in SaTScan.** Analyses were based on residential addresses of cases and controls in 1987 **(3a)** and 1997 **(3b)**. Clusters were found in the Odense (inserted maps) and Copenhagen areas. The colours of the areas indicate the control group used. The number of cases and the *p*-value for each cluster are given in the text boxes. CG: Control Group. The maps contain data from the Danish Geodata Agency and © OpenStreetMap (and) contributors, CC- BY-SA.

Figure 3 shows results of confirmatory analyses performed with SaTScan at two selected points in time 1987 (Figure 3a) and 1997 (Figure 3b) and with each control group and groups combined. In 1987 borderline significant clusters with more than 100 cases covering Copenhagen and its northern suburbs were identified by SaTScan by each control group (red and blue area in Figure 3a). When groups were combined the cluster became statistically significant (yellow area in Figure 3a). The combined control group also yielded a statistically significant cluster in Odense (yellow area in Figure 3a). In 1997 control group 1 and the combined group identified a large and statistically significant cluster in the northern Copenhagen area (red and yellow areas in Figure 3b), while control group 2 identified a cluster in Odense (blue area in Figure 3b). Additional analyses with each of the control groups and the combined group confirmed the cluster area north of Copenhagen in 1977 and at age 50 (results not shown).

**Figure 4:**
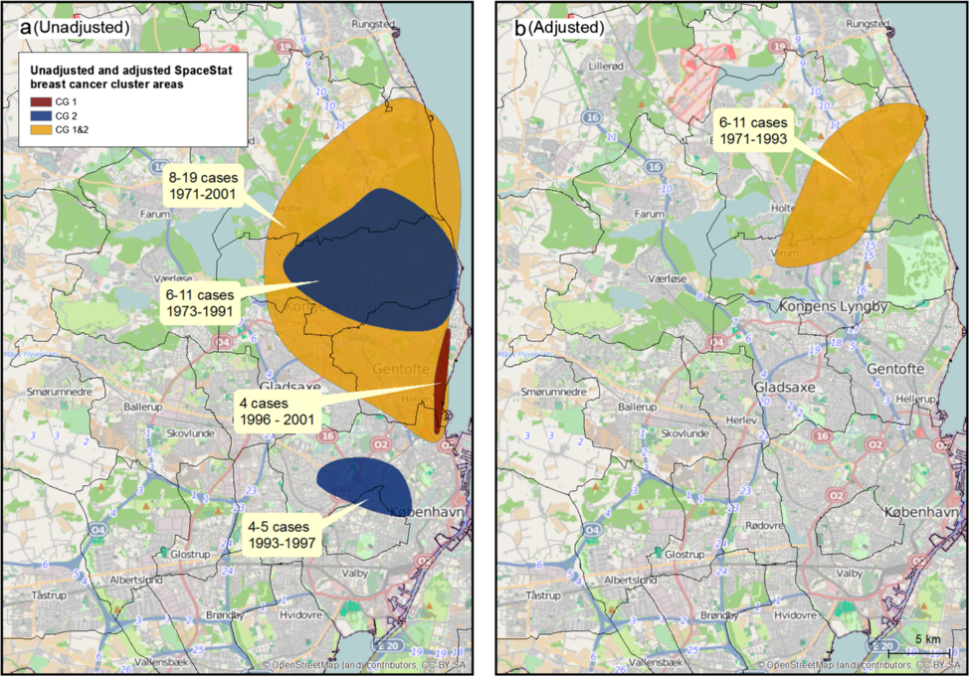
**Unadjusted and adjusted results of space-time cluster analyses.** Analyses were based on the 66% of the study population with data on reproduction and socioeconomic indicators and performed in SpaceStat with 999 permutations, *k* = 100. **4a** shows cluster areas in the Copenhagen areas with each of the two control groups and the combined control group before adjustment. **4b** shows cluster areas detected by identical analyses after adjustment for ever/never child birth, age at first birth, number of child births, area-level income and education. The cluster areas presented in the figures illustrate the maximum extent of the cluster areas based on the location of significant cases, and the colours of the areas indicate the control group used. For each cluster area the text box shows how many cases it comprised and its temporal extent. CG: Control Group. The maps contain data from the Danish Geodata Agency and © OpenStreetMap (and) contributors, CC- BY-SA.

Figure 4 shows results of the unadjusted (Figure 4a) and adjusted (Figure 4b) space-time cluster analyses. In the unadjusted analyses both control groups and the combined group detected time persistent clusters of varying size north of Copenhagen (Figure 4a). Additionally, control group 2 found a small, short term cluster in Copenhagen City (Figure 4a). The cluster north of Copenhagen was confirmed in analyses with other levels of *k* and there was better agreement on the location of the area across the two control groups (results not shown). A small cluster of 1–3 statistically significant cases was detected in Odense with the second and the combined control groups, but it was too small to be regarded a true cluster (not shown).

When analyses were adjusted for reproductive and socioeconomic factors, only the combined control group identified a cluster north of Copenhagen (Figure 4b). The combined control group continued to identify two significant cases in Odense after the adjustment, but applying the control groups separately did not. Findings from the cluster analyses are summarized in table 2, from which it appears that the cluster north of Copenhagen was consistently found across control groups in most analyses, while the Odense and Høje Taastrup areas were detected in fewer analyses and with less agreement.

**Table 2:** Summary of findings from space-time cluster analyses performed in SpaceStat and confirmatory “space-only” cluster analyses performed in SaTScan

**Analysis**		**Identified cluster areas**									**Figure no.**
		**Copenhagen**			**Odense**			**Høje Taastrup**			
***Q*-statistics (SpaceStat)**	*k*^a^	1^b^	2^c^	1 & 2^d^	1	2	1 & 2	1	2	1 & 2	
Unadjusted, all cases											
Calendar year	25	x	x	x		x	x				2.a
	100	x	x	x					x		2.b
Age	25	x									-
	100	x	x	x							-
Unadjusted, 66% of cases											
Calendar year	25	x		x							-
	100	x	x	x							4.a
Age	25										-
	100										-
Adjusted, 66% of cases											
Calendar year	25										-
	100			x							4.b
Age	25										-
	100										-
**Scan statistics (SaTScan)**											
Year 1987		x^e^	x^e^	x			x				3.a
Year 1997		x		x		x					3.b

For each cluster area, the “x” indicates in which analyses the cluster was detected according to method, number of cases, adjustment, time scale, choice of ^a^*k*-nearest neighbours and by control group ^b^1, ^c^2 and ^d^1 & 2 combined. ^e^Only borderline significant. For selected analyses the cluster areas are depicted in the figures listed in the last column.

Finally, 138 cases and 203 controls had resided inside the northern Copenhagen cluster area for at least five years, resulting in a relative risk of breast cancer of 1.39 (95% CI: 1.11-1.74) for women who had lived within the area compared to those who had not. Cases from the cluster area were on average seven years younger and had fever metastases at time of diagnosis than cases living outside the Copenhagen cluster area.

## Discussion

This population-based case–control study consequently found a statistically significant cluster of breast cancer in an area comprising the northern suburbs of Copenhagen present at almost the entire study period. A second cluster was found in Odense; however, this cluster was less evident.

### The northern suburbs of Copenhagen

Clusters in the northern suburbs of Copenhagen were consistently identified spatially and temporally by use of each of the two control groups and when control groups were combined into one. Further, the cluster area was found at several levels of *k* and confirmed by supplementary analyses in SaTScan. The cluster area persisted in crude analyses restricted to the 66% of the study population with data on reproduction and socioeconomic indicators. However, when analyses were adjusted for reproductive and area-level socioeconomic factors the cluster area was smaller and only identified with the combined control group. This could suggest that the clustering of cases in this area is caused by geographical differences in reproductive and/or socioeconomic factors. But as the cluster area did not disappear entirely as a result of the adjustment it is also possible that other factors have contributed to the cluster. Further, with the cluster being persistent when the control groups were combined but not when they were used separately could also indicate that sample size has influenced the results.

### The Odense area

Results also suggested a small cluster of breast cancer cases in Odense; however, this area was only statistically significant when the second and the combined control groups were applied, and when the study population was reduced to 66%, the area only had 1–3 statistically significant cases, which, according to a previous simulation study [27], is too few cases to be regarded a true cluster. This borderline result was further weakened when analyses were adjusted, but it cannot be ruled out that a small cluster existed in this area. On the other hand, as the first control group did not detect this cluster area, it is likely that this finding is merely driven by the geographical pattern of the second control group rather that the cases.

### The Høje Taastrup area

Finally, a cluster was also found in the Høje Taastrup area (south-west of Copenhagen), with the second control group and *Q*-statistics. Results of selected analyses in SaTScan with the second and the combined control group agreed on this area, however since the area was not found by *Q-*statistics with the first or the combined control group nor by SaTScan using the first control group, we regard this a chance finding.

### Time scales

In the space-time cluster analyses we modelled time both as calendar year and as age, because if age-specific susceptibility exists in the development of breast cancer it might be revealed by use of the age time scale [23]. In general, the detected clusters existed for long periods of time in several of the analyses, thus results did not point out any specific time interval for the clusters. However, residential data were not available prior to 1971, thus we did not have information on residential addresses during child- or young adulthood for the majority of the study population, consequently we did not have the possibility to detect clusters that could have occurred during that potentially important period of life.

### Socioeconomic status and breast cancer risk

Breast cancer is one of the few cancers that is associated with high socioeconomic status [10]. However, at the same time affluent women are usually diagnosed at earlier stages and have better survival rates compared to deprived women [30,31]. Although socioeconomic status itself is not regarded a risk factor, its association with breast cancer is thought mediated by other well-established breast cancer risk factors such as high age at first birth, use of HRT and alcohol intake which are frequent among women with high socioeconomic status compared to less affluent women [32]. As the suburbs north of Copenhagen are characterised by a wealthy and highly educated population, it seems plausible that the cluster in this area could be explained by factors related to high socioeconomic status. This also agrees with our finding of younger age at diagnosis and lower frequency of metastasis among cases, who lived inside the cluster area compared to those who lived outside. The fact that the cluster area mostly disappeared after adjustment for reproductive factors and area-level income and education (the cluster area was only detected with the combined control group) supports this hypothesis. On the other hand, the area persisted to have a smaller significant cluster when the combined control group was used, which could also suggest that other factors may have contributed to the observed breast cancer cluster. Data from the Danish National Health Survey 2010, including 177,639 responders, show that the municipalities north of Copenhagen have some of the highest proportions of women with potential problematic alcohol consumption compared to the rest of the country [33]. Further, the Danish prescription database indicates that the HRT use might be slightly higher in the capital region than in the remaining four Danish regions [34], however the differences are small and numbers are aggregated to very large geographical units. Nevertheless, it seems possible that alcohol and/or HRT could have contributed to the observed breast cancer cluster.

Previous studies have found that high socioeconomic status at the individual- and at the area-level independent of each other were associated with higher risk of breast cancer [35,36]. The area-level socioeconomic adjustment performed in the present study would therefore have been improved if we had also been able to adjust for individual-level socioeconomic factors.

Finally, we cannot exclude that other factors (e.g. environmental) with geographical variation might have contributed to the observed cluster. Since the cluster persisted through time, the responsible factor(s) are likely to have similarly persisted across many years. The area north of Copenhagen, where the cluster was detected, is mainly a residential area with single family houses, green spaces, forests and lakes. The area has lower population density than Copenhagen City, but higher than in municipalities further away from Copenhagen. Farming and heavy industry is not present in the area; but a highway and some major roads, railways and power lines intersect the area. However, large parts of Denmark have such infrastructure, therefore it seems unlikely that these factors could be related to the clustering of cases.

### Previous cluster studies of breast cancer

Several previous studies have identified clusters of breast cancer, however it is difficult to compare these to the present study because they were conducted in other populations (in the US and Canada) and they use different methods and types of data. One previous study found high breast cancer mortality in a large region covering New York and Philadelphia metropolitan areas, using the scan statistic of SaTScan [16]. Applying a different approach (Moran’s I) and focusing on Long Island only, Jacquez and Greiling identified local clusters of high breast cancer morbidity rates in the Southampton area [37]. Both studies relied on aggregated data, thus they were unable to take human mobility and potential latency periods into account. Further, clusters of mortality (in contrast to incidence) might reflect differences in cancer treatment and survival. Studying clustering of breast cancer in two New York state counties, Han et al. applied different cluster detection techniques to the spatial pattern described by place of residence at several selected points in time over the life-course of breast cancer cases and controls, and found clustering of pre-menopausal breast cancer cases’ residential addresses at time of birth and at time of menarche [18,19]. Although the study was based on residential histories and attempted to identify susceptible time periods related to breast cancer development, it only investigated the spatial distribution of cases and controls at a few selected points in time over the life-course.

A recent Canadian study applied the scan statistics of SaTScan and found excess incidence rates of breast cancer in five counties in southern Ontario, which were suggested related to environmental pollution from industry and farming characterising these areas [17]. The study was based on aggregated incidence data and results should therefore be interpreted with caution. A study on Cape Cod, Massachusetts, however, acknowledged that exposures at past residencies rather than exposures at time of diagnosis may be more relevant in the development of breast cancer. Thus, the study was based on 40 years of residential histories and used generalised additive models to identify clusters of breast cancer in space and time simultaneously, adjusting at the same time for known risk factors such as parity and age at first birth. A large area of elevated breast cancer risk was found near Massachusetts Military Reservation in the 1940s and 1950s (many years before cases were diagnosed), suggesting that activities at this site in that time window could have exposed women living close by to hazardous substances [20,38]. In contrast to these previous studies on Cape Cod, results of our present study do not point to specific environmental causes, rather it suggests that the cluster north of Copenhagen may be a result of already established factors related to high socioeconomic status. There was no organised breast cancer screening programme established in the municipalities north of Copenhagen at the time when cases of the present study were diagnosed, but we cannot exclude that affluent women are more likely to seek breast cancer screening on own initiative than deprived women, which could have contributed to the observed cluster.

### Strengths and limitations

The present study is among the first examinations of clusters of breast cancer in both space and time using residential histories. Cases were identified in the virtually complete high-quality population-based Danish Cancer Registry [21,39], thus the study had very reliable case ascertainment. Furthermore, the Danish Civil Registration System provided an ideal frame for bias-free control selection and collection of residential addresses back to 1971 [22]. Compared to other case–control studies that usually have to rely on residential histories collected by interview, our register-based residential histories strengthened the study. The advantageous study design with two independent control groups and the ability to adjust for reproductive factors and area-level socioeconomic indicators was very helpful in the interpretation of the findings. Further, the scan statistics of SaTScan confirmed the location of the cluster areas. In a previous study of non-Hodgkin Lymphoma using the same study design, there was no consistent finding across the two independent control groups and combining the control groups into one made the clusters disappear, leading to the conclusion that there were no space-time clusters of incident non-Hodgkin Lymphoma cases based on residential histories in Denmark [40]. However, this was not the case in the present study of breast cancer, where we were able to replicate within-study findings across control groups.

The lack of data on alcohol intake and use of HRT limits our ability to interpret the likely causes of the cluster in our study, as these known risk factors may explain at least part of the detected cluster; however the inability to adjust for all known risk factors is a shortcoming of almost all cluster studies. Due to no more than 33 years of residential history data we did not have the possibility to detect clusters that could have occurred early in life or young adulthood, which is an important limitation of the study. Furthermore, seven percent of the residential addresses were geocoded at the municipality level, which could have introduced some uncertainty to the study; however sensitivity analyses that omitted these less precise addresses indicated that results were not influenced by the geocoding uncertainty. Another limiting factor was related to computational time. Due to the large data sets of residential histories, a single analysis took up to 8 hours, thus we could not explore a very wide range of different levels of *k* in the analyses. But SaTScan spatial scan statistics, which search for clusters using variable-sized scanning windows, were used to confirm the clusters suggested by the *Q*-statistics. The *Q*-statistics employ local space-time statistics for each individual and the question of inference with multiple tests arises. To address multiple testing we used two approaches driven by our simulation study [27]. The first was to combine information from two *Q*-statistics, such that a possible true cluster warrants further investigation if four or more significant cases were detected in the same area with a *Q*_*i*_*p* = 0.001 and *Q*_*it*_*p* ≤ 0.05. The second was to use SaTScan to corroborate clustering in the time periods found significant under the *Q*-statistics. While not strictly a multiple testing correction, using SaTScan to corroborate results increased our confidence in the finding provided by the *Q*-statistics, further reducing the possibility of a false finding due to multiple testing.

## Conclusions

The present study found a space-time cluster of breast cancer in the municipalities north of Copenhagen based on 33 years of residential histories. The cluster was consistently found across two independent control groups, but after adjustment for reproductive factors and area-level income and education the cluster became less evident. The remaining less evident cluster might be explained by socioeconomic factors that were not accounted for such as individual-level income and education, alcohol consumption and HRT use. We cannot exclude environmental pollutants as a contributing cause, but no pollutants specific to this area seem obvious.

## Abbreviations

HRT: Hormone replacement therapy; CG: Control group.

## Competing interests

Geoffrey M. Jacquez has an interest in BioMedware, the developer of the SpaceStat software used in this study. This has not influenced interpretation of the results and does not alter the authors’ adherence to all of the policies of BMC Cancer. The authors declare that they have no further competing interests.

## Authors’ contributions

All authors have contributed to study design and interpretation of results. RBN preformed data collection, carried out the analyses, and wrote the paper. GMJ and JRM developed the method and provided analysis tools. AHP, JRM, AKE, GMJ and ORN critically revised the manuscript. All authors have read and approved the final manuscript.

## Pre-publication history

The pre-publication history for this paper can be accessed here:

http://www.biomedcentral.com/1471-2407/14/255/prepub
